# 

**Published:** 1897-09

**Authors:** 


### CONTENTS.

ORIGINAL COMMUNICATIONS. PAGE

Cataphoresis. By Louis Jack, D.D.S. . . . . . . . . . . . . . . . . . . . . . 549
President's Address to the American Dental Association, 1897. By James Truman, D.D.S. . . . . . . . . . . . . . . . . . . . . . . . . . . . . . 563
Ancient Metallurgy. By Henry Leffmann, M.D., D.D.S. . . . . . . . . . . 574
The Study of Anatomy. By W. C. Barrett, M.D., D.D.S., M.D.S., Buffalo, N. Y. 581

REPORTS OF SOCIETY MEETINGS.

American Dental Association . . . . . . . . . . . . . . . . . . . . . . . 586
The New York Institute of Stomatology . . . . . . . . . . . . . . . . . 588
Odontological Society of Pennsylvania . . . . . . . . . . . . . . . . . . 598
National Association of Dental Faculties . . . . . . . . . . . . . . . . . 605

EDITORIAL.

Old Point Comfort . . . . . . . . . . . . . . . . . . . . . . . . . . . . 611
The Antagonism of Law . . . . . . . . . . . . . . . . . . . . . . . . . . 614

BIBLIOGRAPHY . . . . . . . . . . . . . . . . . . . . . . . . . . . . . . 616

OBITUARY.

Dr. Samuel J. Hayes . . . . . . . . . . . . . . . . . . . . . . . . . . . 623

NOTES AND COMMENTS . . . . . . . . . . . . . . . . . . . . . . . . . . 624

CURRENT NEWS.

New Jersey State Dental Society . . . . . . . . . . . . . . . . . . . . . 624

